# Integrated Data Management for Clinical Studies: Automatic Transformation of Data Models with Semantic Annotations for Principal Investigators, Data Managers and Statisticians

**DOI:** 10.1371/journal.pone.0090492

**Published:** 2014-02-28

**Authors:** Martin Dugas, Susanne Dugas-Breit

**Affiliations:** 1 Institute of Medical Informatics, University of Münster, Münster, Germany; 2 Klinik und Poliklinik für Dermatologie und Allergologie, Ludwig-Maximilians-Universitaet, Munich, Germany; The Cochrane Collaboration, Germany

## Abstract

Design, execution and analysis of clinical studies involves several stakeholders with different professional backgrounds. Typically, principle investigators are familiar with standard office tools, data managers apply electronic data capture (EDC) systems and statisticians work with statistics software. Case report forms (CRFs) specify the data model of study subjects, evolve over time and consist of hundreds to thousands of data items per study. To avoid erroneous manual transformation work, a converting tool for different representations of study data models was designed. It can convert between office format, EDC and statistics format. In addition, it supports semantic annotations, which enable precise definitions for data items. A reference implementation is available as open source package ODMconverter at http://cran.r-project.org.

## Introduction

Several stakeholders are involved in clinical trials, in particular principal investigators (PIs), data managers and statisticians. Based on their medical background, principal investigators describe informally what data need to be collected to fulfill the study objective. This informal description of a data model is discussed and refined together with data managers and statisticians. The result of this iterative and interactive process between principal investigators, data managers and statisticians is a set of case report forms (CRFs) for each study. From an informatics point of view, these CRFs specify the data model of study subjects. All data items in those CRFs need to be well defined, including permissible values for each item.

Data models in clinical studies are increasingly complex. Since introduction of the European Clinical Trials Directive (2001/20/EC), the average length of CRFs increased from 55 pages (1999–2002) to 180 pages (2003–2006) per trial [1], associated with major additional costs [2]. Under the assumption that a typical CRF page contains 20–50 items, this corresponds to 3600 to 9000 data items per trial. Obviously, the number of data items is associated with the amount of data management work, which is one of the major cost factors in clinical trials [3]. CRFs define what data will be collected for the study and therefore determine what data items are available for statistical analysis at the end of the study. For design, execution and analysis of a study different representations of the data model are needed. In the design phase, PIs typically apply standard office tools (like word processing or spreadsheet programs) to describe what kind of data need to be collected for a study. For study execution, data managers are working with electronic data capture (EDC) systems and statisticians apply dedicated statistical software for data analysis. Therefore in a typical study setting at least three different representations of the data model are created and need to be updated continuously: The study data model in office format, EDC and statistics format.

The **objective** of this work is to develop and assess automated methods to transform data models suitable for principal investigators, data managers and statisticians. These transformations should preserve the semantics of the data model, therefore semantic annotations should be included in this transformation process.

## Methods

### Data Models for Data Management in Clinical Studies

Electronic Data Capture (EDC) systems are applied to provide electronic case report forms (eCRFs). These systems are customized by data managers for each clinical study. EDC systems for clinical trials need to be validated according to regulations from U.S. Food and Drug Administration (FDA) [4] and European Medicines Agency (EMA) [5]. In cooperation with FDA and EMA the Clinical Data Interchange Standards Consortium (CDISC) defined the operational data model (ODM) [6], an international, open standard for metadata and data in clinical studies. CDISC ODM is supported by many commercial EDC systems, for example medidata Rave(R) [7], XClinical Marvin [8] and secuTrial(R) [9]. In addition, CDISC ODM can be semantically annotated [10]. For these reasons an automated transformation method for data models in clinical studies should be able to process data models in ODM format. Consequently, CDISC ODM was selected as data format for EDC systems in the reference implementation.

### Statistics Software in Clinical Studies

At present, SAS [11] and IBM SPSS [12] are commonly used commercial software packages for data analysis in clinical studies. To enable wide spread use of a reference implementation, an open source system is preferable. R [13] is the open source version of S-Plus [14], another well-known statistical software tool. R enables to export/import datasets to/from IBM SPSS and SAS. Therefore R was chosen to represent the study data model for statistical analysis. The data model is represented by an R data frame.

### Semantic Annotations for Data Models

Data models for study subjects are defined by CRFs. Each CRF consists of data items (for example “patient gender”), which can be organized in item groups (for example “demographics”). Each data item is associated with a set of permissible values (for example “male”, “female”). In principle, at each level semantic annotations from healthcare terminologies can be added to define the semantics of data items, item groups and permissible values.

These annotations can help to overcome the ambiguities of natural language and enable more precise specifications of data models. Item names can be ambiguous, for example “length” can refer to length of an arm or length of a leg; abbreviations can be plurivalent, for instance “MS” can denote multiple sclerosis or mitral stenosis; some data items can be determined in different ways, e.g. blood pressure can be measured in different positions (sitting, lying, etc.) and with different methods (non-invasive, invasive). Semantic codes can provide references to detailed specifications of data items, both regarding medical concepts (what is the contents of this item?) and permissible values for each item. In addition, semantic codes can be directly processed by computer programs and used for comparisons and transformations of data models. Each semantic annotation consists of a terminology version and an associated code value.

SNOMED CT [15] is a commonly used healthcare terminology, which can be applied for semantic annotations. SNOMED CT codes are characterized by a certain terminology version (for example “SNOMED CT 2010_0731”) and a code value (for instance “248153007” to represent “male”). Logical Observation Identifiers Names and Codes (LOINC(R)) [16] is another code system which can be applied for semantic annotations of data models. In particular, LOINC(R) provides a large variety of codes regarding laboratory procedures. The Unified Medical Language System (UMLS(R)) [17] is a Metathesaurus consisting of terms and codes from more than 100 different healthcare terminologies. Therefore the UMLS provides a unique richness of semantic codes (>1.4 Mio. concept codes as of July 2013).

### Data Model Transformation

To transform data models between the various representations (EDC, statistics, office), R [13] functions were designed as public reference implementation. These programs contain parsers for the different formats with file-based input and output. Regarding the office format, a specific Microsoft Excel template was designed to capture semantic annotations (also available in csv-format for portability). In principal, this reference implementation can be used with any medical terminology consisting of terms and associated codes.

### Evaluation Approach

If an automated transformation of study data models in office format, EDC and statistics format is feasible, then transformation from EDC into statistics format and back again into EDC format should result in the same EDC representation.

Similarly, transformation from EDC format into office format and back again into EDC format should result in the same EDC representation. In particular, semantic annotation at the various levels (itemgroup, item, permissible values) should be preserved.

This evaluation procedure was applied to a simple data model with few data items and then to a random sample of 10 real-world ODM files from a public portal for medical data models in ODM format [18]. As a third evaluation step, the transformation from office into EDC format was tested for approximately 400 forms from clinical trials.

## Results

### Reference Implementation for Study Data Model Transformations

A reference implementation for automatic transformation of data models with semantic annotations for principal investigators, data managers and statisticians was developed. It is implemented in R and available as open source package ODMconverter at http://cran.r-project.org. This software enables to transform a data model for study subjects back and forth between different representations: office format for principal investigators, EDC format for data managers and statistics format for statisticians. Semantic annotations are preserved by these transformations.

Table 1 presents a simplified example of a data model in office format. It is a simple spreadsheet which contains few administrative information about the study and then basically a catalogue of data items. The description and selection of relevant data items requires medical expertise, therefore this representation of the data model needs to be editable by medical personnel without special computer skills. In addition to item descriptions also semantic codes can be provided. These codes can be looked up with various tools, for instance using the NCImetathesaurus [19]. Again, selection of appropriate semantic codes from healthcare terminologies like SNOMED CT requires medical expertise and cannot be done by data managers or statisticians alone.

**Table 1:** Simplified example of data model in office format (spreadsheet).

	A	B	C	D	E	F
1	StudyOID	S.0000				
2	Sponsor	Testsponsor				
3	Condition	Testcondition				
4	StudyName	ODM Test Study				
5	StudyDescription	Test of ODM tools				
6	Form	ODM-Test				
7	FirstName	Test				
8	LastName	Testname				
9	Organization	Test organization				
10						
11	Type	Name	en	UMLS CUI	SNOMED CT 2010_0731	LOINC
12	itemgroup	Info	General Information	C0332118	106227002	
13	boolean	Willingness	Willingness to participatein clinicialtrials	C1516879		
14	integer	Age	Age		102518004	
15	date	DOB	Date of Birth		152322001	
16	integer	Gender	Gender		139865004	
17	codelistitem	1	male	C0024554	248153007	
18	codelistitem	2	female	C0015780	248152002	
19	string	DiagnosisTx	Diagnosis text		439401001	
20	string	DiagnosisCd	Diagnosis code			
21	float	Crea	Creatinine			38483–4
22	time	labTime	Time of lab value			

The header (line 1–9) contains general information about the study. Line 13–22 provide data items of different data types (column A). Column C presents item labels (en = english). Columns D,E,F contain semantic codes for each data item.

When a study protocol is completed and approved, the study database needs to be implemented. CDISC ODM is an open standard for study data models and endorsed by regulatory agencies, therefore it was chosen as EDC format in the reference implementation. The software package ODMconverter provides a function office2ODM which converts the format presented in table 1 into ODM format. ODM files can be directly imported into several available EDC systems to setup the study database.

**Figure 1:**
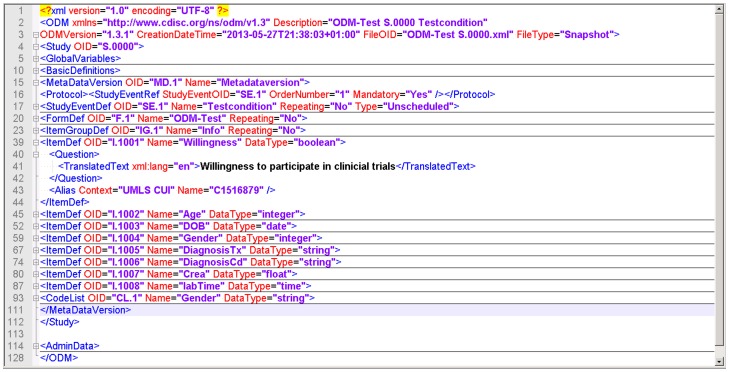
Example of data model in CDISC ODM-format. It consists of one form (“ODM-Test”) with one itemgroup (“Info”) and 8 data items. Details for item I.001 are displayed, including item name, detailed description in english and its associated UMLS code.

**Figure 2:**
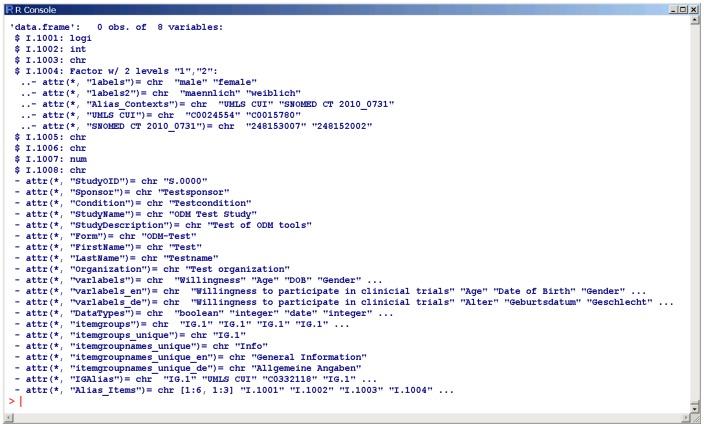
Example of data model in statistics format. An R data frame is provided with 8 variables (I.001 … I.008). Labels for variables and permissible values are defined, for instance “male” and “female” for item I.1004 (Gender). General information about the study like “StudyName” is provided as attribute of this data frame.

When the data collection of a study is completed and all activities to achieve high data quality are finished, the database is closed and the data set is handed over to a statistician. The data set needs to be transferred from the EDC system into a statistical software package. At this point, a transformation of the study data model from EDC format (Figure 1) into statistics format is required. The software package ODMconverter provides a function ODM2R for this task. Figure 2 presents the transformation result of the data model from Figure 1 into an R data frame. As a specific feature all semantic annotations from previous steps are preserved.

These data model transformations can be inverted, again with preservation of semantic annotations. For this purpose, package ODMconverter provides functions R2ODM and ODM2office.

### Evaluation

As a first evaluation step, a simple data model with 8 data items was converted from office format into ODM, then into an R data frame, then back into ODM and finally into office format. All intermediate files were checked and verified manually.

As a second evaluation step, this data model conversion process was applied to a random sample of 10 “real-world” ODM-files from a public ODM-Portal [20], see Table 2.

**Table 2:** Trial IDs, medical condition, number of items and number of annotation codes regarding 10 forms in ODM format, which were used for the evaluation (randomly selected from www.medical-data-models.org).

Trial ID	Medical Condition	Number of items	Number of annotation codes
NCT00824083	Ewing-Sarcoma	5	44
NCT00980135	Atopic Dermatitis	12	135
NCT01104584	Breast Cancer	17	219
NCT01147939	Acute Myeloid Leukemia	27	306
NCT01179620	Renal Dialysis	8	53
NCT01283724	Endometriosis	11	172
NCT01324947	Multiple Myeloma	33	333
NCT01361334	Acute Myeloid Leukemia	28	376
NCT01403376	Multiple Sclerosis	16	163
NCT01408095	Diabetes Mellitus, Type 2	27	355

These 10 files in ODM format were converted into csv(comma separated value)- format (function ODM2office) and then back into ODM format (function office2ODM). All 10 files were transformed into an R data frame (function ODM2R) and then back into ODM format (function R2ODM). All intermediate files were checked and verified manually using a standard text editor (Notetab++ version 5.6.8) and Microsoft Excel (version 2010). A recently developed method to automatically compare medical forms [21] was applied to verify that all generated ODM files contained identical items and semantic annotations.

As a third step, the transformation from office into EDC format was tested for a larger set of forms from clinical trials. Approximately 400 eligibility forms from clinical trials with active participation from Münster University Hospital were identified in the Internet [22]. These forms were manually annotated with semantic codes, in particular UMLS and SNOMED CT codes using Microsoft Excel templates. Using ODMconverter, these files were converted into ODM format and then uploaded into an Internet portal [20].

## Discussion

With the proposed reference implementation and its technical evaluation we demonstrated that automatic transformation of data models with semantic annotations for principal investigators, data managers and statisticians is feasible. We did a literature search (PubMed, Google) and were not able to identify a similar approach. This method of integrated data management is currently being applied in practice to design and implement an observational study regarding craniocerebral injuries in Münster, Germany.

In general, stakeholders with different professional backgrounds need to work together in clinical studies. Data management for studies consumes a lot of resources [2] and requires contributions from principal investigators, data managers as well as statisticians. A key task is to design and implement a set of CRFs for each study. Currently, different tools are being applied for this task, in particular office tools like Microsoft Excel, EDC tools and statistics software. In clinical trials, CRFs are quite complex with 180 CRF pages on average [1]. Given this complexity of data models, the iterative nature of CRF design, and the need to synchronize different representations (office/EDC/statistics format), an automated method obviously can help to reduce manual, error-prone transformation work. In contrast to generic extract-transformation-load (ETL) tools, no customization of an ETL process is needed with our method, because it is based upon CDISC ODM.

Another important aspect of the proposed method is semantic annotation of data models. Design, execution and analysis of clinical studies involves several stakeholders with different backgrounds. Despite the availabilty of international healthcare terminologies like SNOMED CT and LOINC for many years, these are currently used only very rarely in clinical studies. In regulated trials a lot of coding is done with the Medical Dictionary for Regulatory Activities (MedDRA) [23], but MedDRA codes are typically applied to item values, not to items themselves. Unfortunately, common statistics programs like IBM-SPSS currently do not yet provide semantic annotation functions. The key advantage of semantic annotations is a precise specification of items. By this means ambiguities about the meaning of CRF items can be avoided for all stakeholders. Item names in free text can be ambiguous: “size” can be size of the patient or size of its tumor, a lab value “creatinine” can refer to serum or urine concentration, blood pressure can refer to arterial or venous pressure. In clinical trials this issue of the precise meaning of items is addressed by the study protocol and standard operating procedures (SOPs). Semantic codes can provide computable references to precise medical definitions and thereby contribute to shorter and more concise SOPs. For example, a blood pressure finding in sitting position can be specified by UMLS code C1271104 (blood pressure finding) and UMLS code C0277814 (sitting position). A key aspect of the proposed reference implementation is preservation of these semantic annotations during all transformation steps.

So far, the proposed method was mainly evaluated from a technical perspective. It was not yet formally validated. This approach is focussed on data structures and does not provide a complete specification of the study data model. However, is the current manual transformation process being validated regularly in clinical studies? It seems quite unlikely that three different representations of a data model in several versions are fully synchronized in a setting with thousands of data items.

Future work will need to address application of this transformation method in clinical study settings to assess its benefits.

## Conclusion

Automated transformation of semantically enriched medical data models between office format, CDISC ODM and statistics format is feasible.
